# Crystal structure, Hirshfeld surface analysis and DFT studies of 4-amino-*N*′-[(1*E*)-1-(3-hy­droxyphen­yl)ethyl­idene]benzohydrazide

**DOI:** 10.1107/S205698902500297X

**Published:** 2025-04-08

**Authors:** Subramani Uma Maheswari, Srinivasan Senthilkumar, Sivashanmugam Selvanayagam

**Affiliations:** a Department of Chemistry Annamalai University, Annamalainagar Chidambaram 608 002 India; b Department of Science and Humanities, Dhaanish Ahmed Institute of Technology, Coimbatore 641 042, India; c PG & Research Department of Physics, Government Arts College, Melur 625 106, India; Vienna University of Technology, Austria

**Keywords:** crystal structure, benzohydrazide derivative, N—H⋯O and O—H⋯O inter­molecular hydrogen bonds, Hirshfeld surface analysis, DFT analysis

## Abstract

In the title compound, C_15_H_15_N_3_O_2_, (**I**), the aniline and phenol rings form a dihedral angle of 62.1 (1)°. Inter­molecular N—H⋯O and O—H⋯O hydrogen bonds lead to the formation of sheets extending parallel to (010). Inter­molecular inter­actions were qu­anti­fied and analysed using Hirshfeld surface analysis, revealing that H⋯H inter­actions contribute most to the crystal packing (42.2%). The mol­ecular structure was optimized by density functional theory (DFT) at the B3LYP/6–31 G(d,p) level and was compared with the experimentally determined mol­ecular structure in the solid state.

## Abstract 2

In the crystal structure of the title compound, C_15_H_15_N_3_O_2_, O—H⋯O and N—H⋯O hydrogen bonds lead to the formation of layers extending parallel to (010).

## 1. Chemical context

Hydrazones have been found to show various biological properties, including anti­oxidant (Belkheiri *et al.*, 2010 ▸), anti-inflammatory (Radwan *et al.*, 2007 ▸) and anti­cancer (Kumar *et al.*, 2012 ▸) effects.
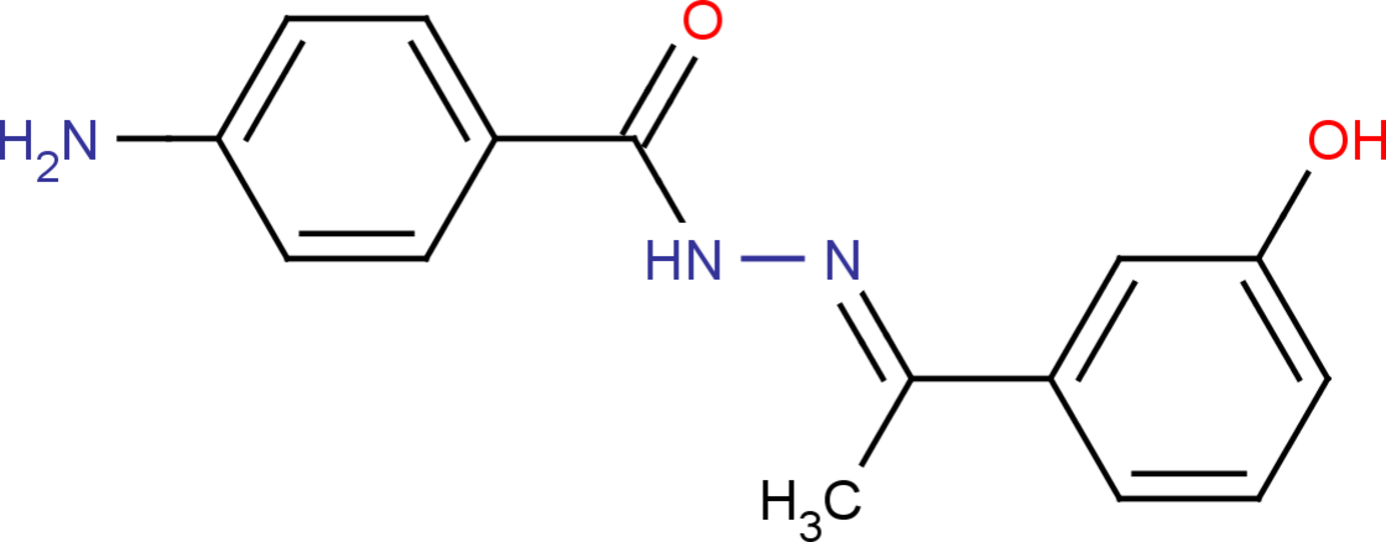


In the present work, the synthesis, structural and computational studies of another hydro­zone, 4-amino-*N*′-[(1*E*)-1-(3-hy­droxy­phen­yl)ethyl­idene]benzohydrazide, (**I**), is reported.

## 2. Structural commentary

**Figure 1:**
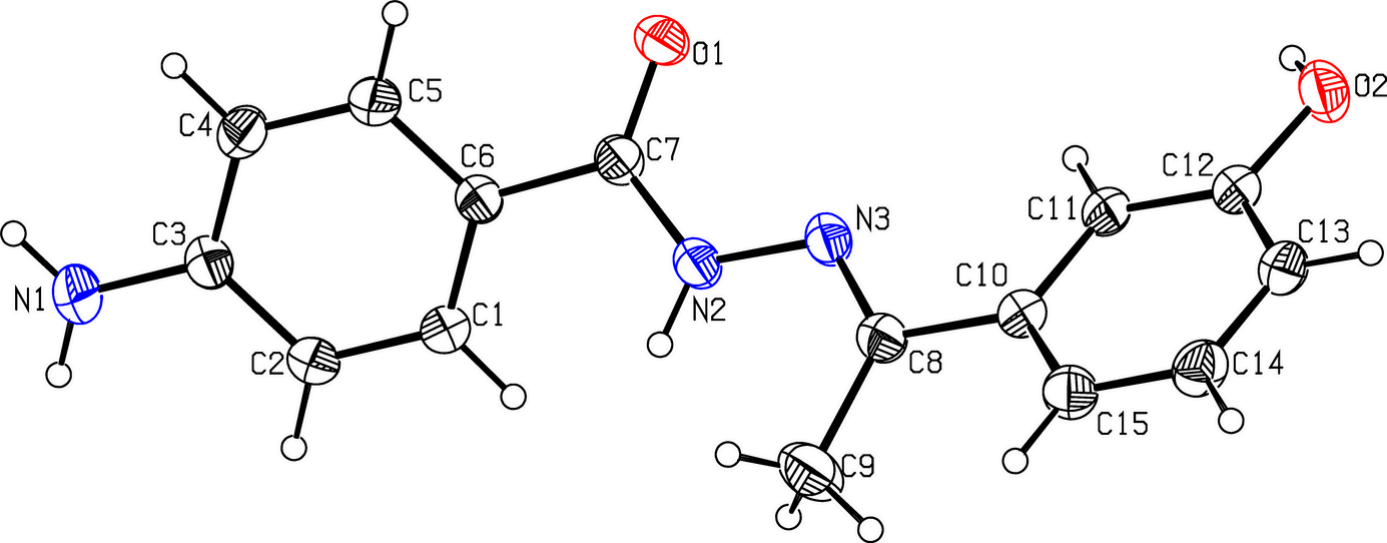
The mol­ecular structure of (**I**) with displacement ellipsoids drawn at the 30% probability level.

The mol­ecular structure of (**I**) is displayed in Fig. 1 ▸. The aniline ring (C1–C6/N1) is planar with a maximum deviation of 0.023 (1) Å for atom N1. Likewise, the phenol ring (C10–C15/O2) is planar with a maximum deviation of 0.003 (2) Å for atom C12. These two rings are oriented at a dihedral angle of 62.1 (1)°. The least-squares plane calculation of the *N*′-[(1*E*)-ethyl­idene]formohydrazide moiety (C7/O1/N2/N3/C8/C9) reveals that this part of the mol­ecule is nearly planar with a maximum deviation of −0.223 (1) Å for atom O1. This moiety forms dihedral angles of 36.5 (1) and 25.6 (1)°, respectively, with respect to the aniline and phenol rings.

## 3. Supra­molecular features

**Figure 2:**
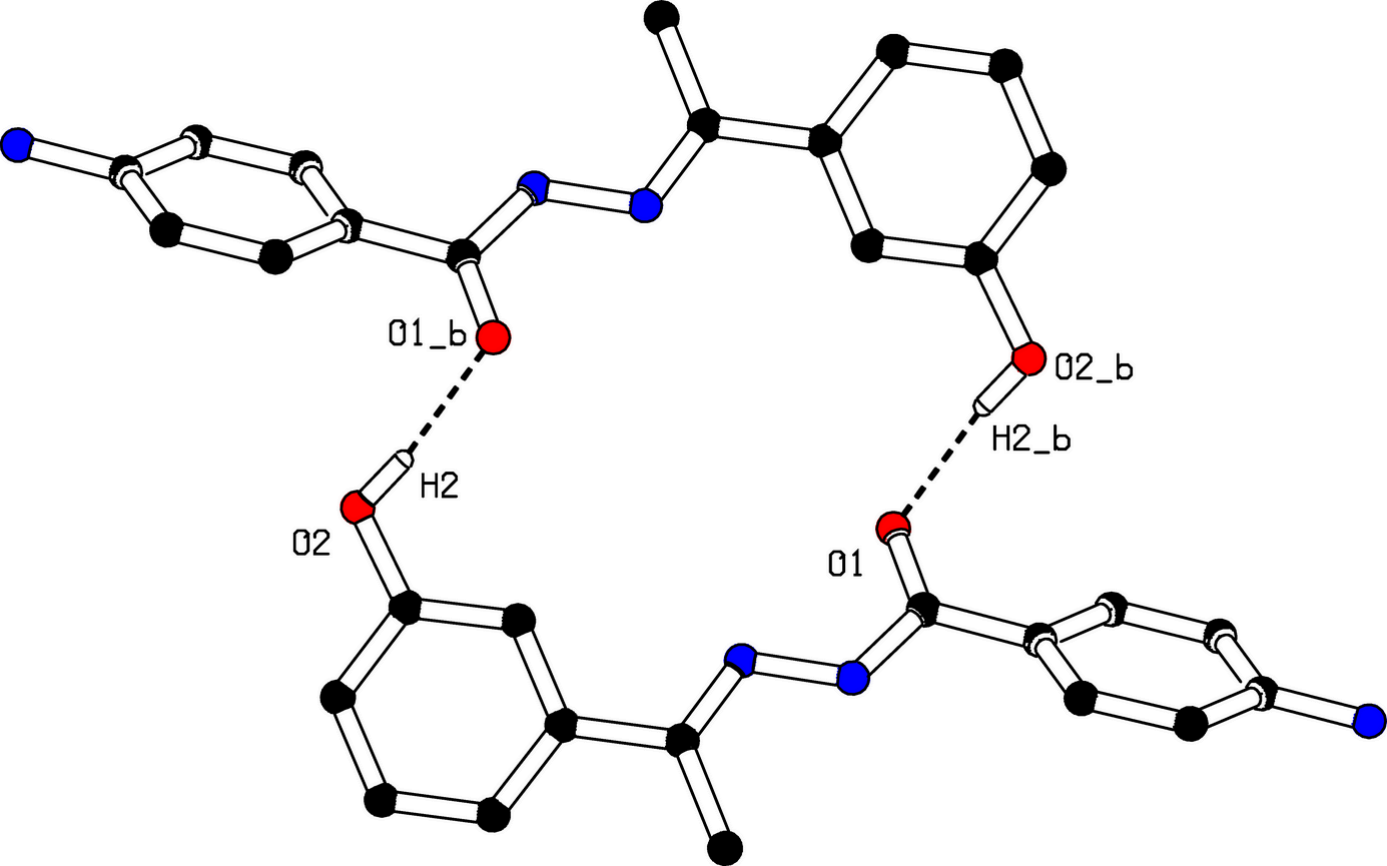
The formation of a centrosymmetric dimer in the crystal structure of (**I**) through O—H⋯O hydrogen bonds. [Symmetry code: (*b*) −*x* + 1, −*y* + 2, −*z*.]

**Figure 3:**
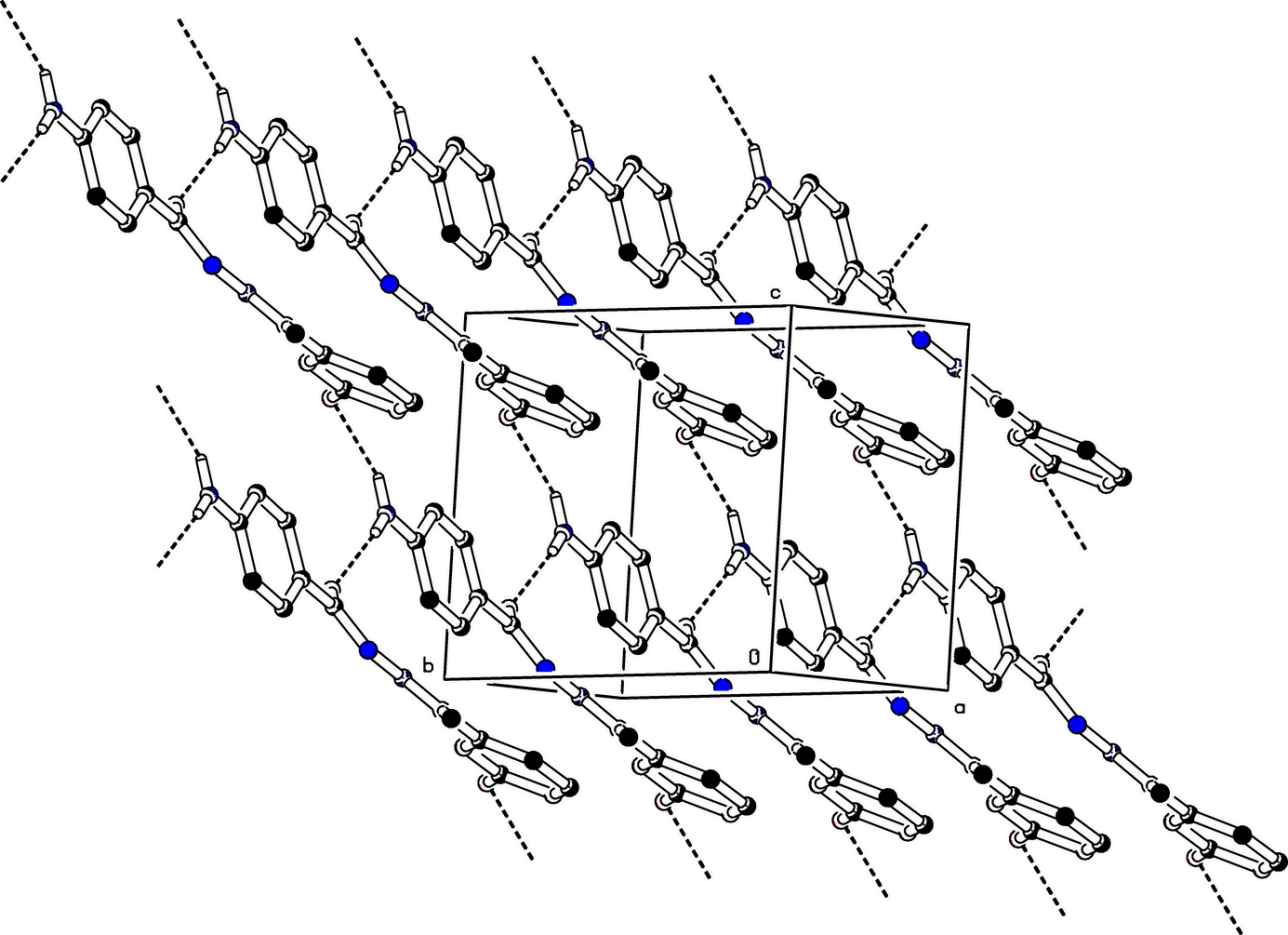
Inter­molecular N—H⋯O and O—H⋯O hydrogen bonds in (**I**) shown as dashed lines. For clarity, H atoms not involved in these hydrogen bonds have been omitted.

In the crystal, mol­ecules associate pairwise *via* O2—H2⋯O1^i^ hydrogen bonds (Table 1 ▸) into inversion dimers with an 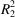 (20) graph-set motif (Etter *et al.*, 1990 ▸), as shown in Fig. 2 ▸. Mol­ecules are further linked into a *C*(14) chain motif by N1—H1*A*⋯O2^iii^ hydrogen bonds running parallel to [100], and by N1—H1*B*⋯O1^ii^ hydrogen bonds into a *C*(8) chain motif running along [102] (Table 1 ▸; Fig. 3 ▸). Taken together, these inter­actions lead to a layered arrangement parallel to (010). It is inter­esting to note that the amine function (N2—H2*A*) is not involved in any inter­molecular inter­actions.

## 4. Hirshfeld surface analysis

**Figure 4:**
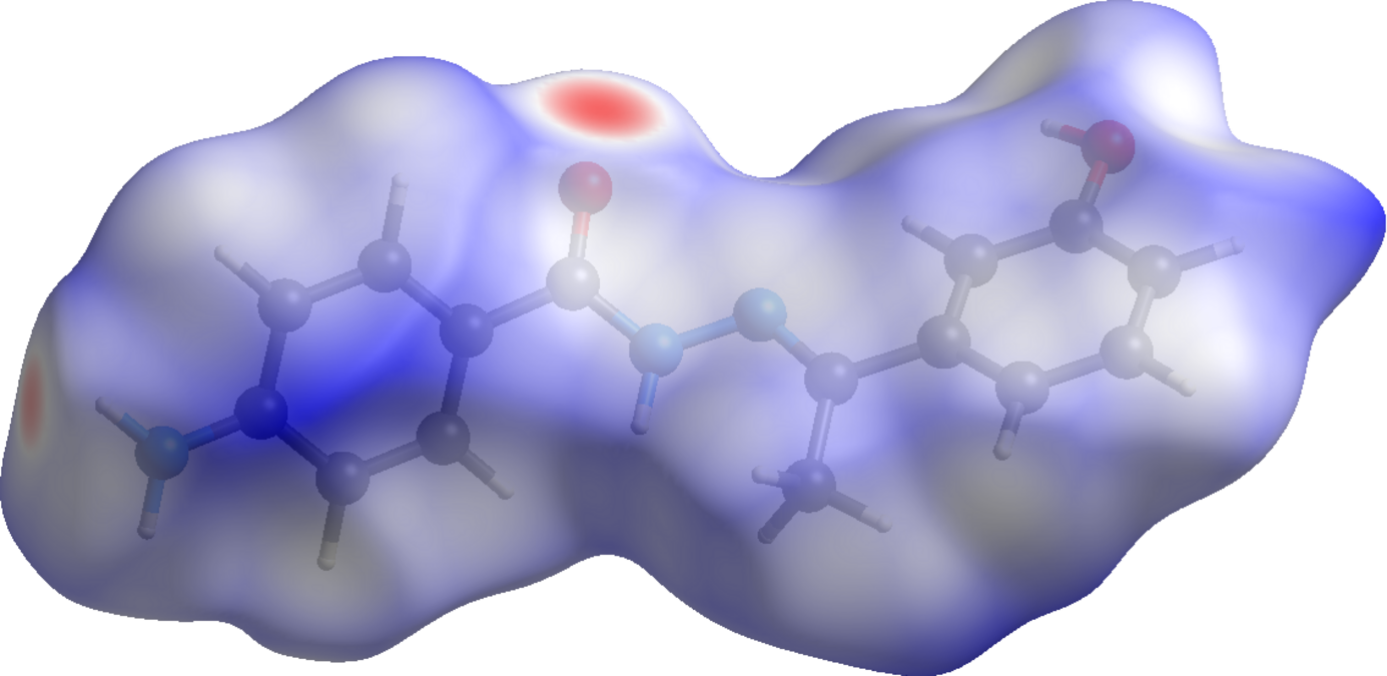
The Hirshfeld surface mapped for (**I**) over *d*_norm_.

To further characterize the inter­molecular inter­actions in (**I**), a Hirshfeld surface (HS) analysis (Spackman & Jayatilaka, 2009 ▸) was carried out using *CrystalExplorer* (Spackman *et al.*, 2021 ▸). The HS mapped over *d*_norm_ is illustrated in Fig. 4 ▸, showing the aforementioned hydrogen-bonding inter­actions as red-colored areas.

**Figure 5:**
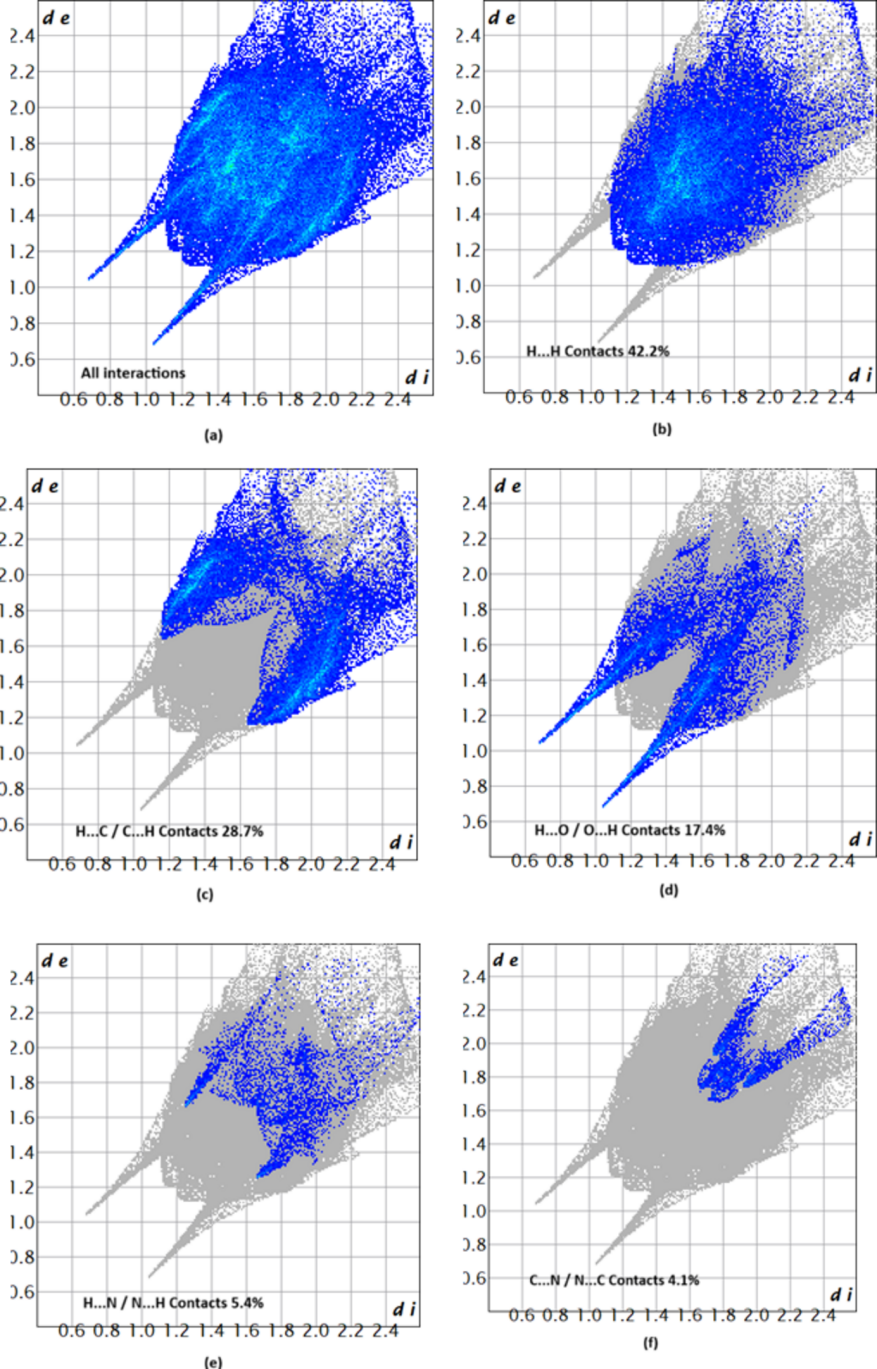
Two-dimensional fingerprint plots for (**I**), showing (*a*) all inter­actions, and delineated into (*b*) H⋯H, (*c*) H⋯C/C⋯H, (*d*) H⋯O/O⋯H, (*e*) H⋯N/N⋯H and (*f*) N⋯C/C⋯N inter­actions with their relative contributions. The *d*_i_ and *d*_e_ values are the closest inter­nal and external distances (in Å) from given points on the Hirshfeld surface.

The associated two-dimensional fingerprint plots (McKinnon *et al.*, 2007 ▸) provide qu­anti­tative information about the non-covalent inter­actions in the crystal packing in terms of the percentage contribution of the inter­atomic contacts (Spackman & McKinnon, 2002 ▸). The overall two-dimensional fingerprint plot is shown in Fig. 5 ▸*a*. The HS analysis reveals that H⋯H and H⋯C/C⋯H contacts are the main contributors to the crystal packing, followed by H⋯O/O⋯H, N⋯H/H⋯N and C⋯N/N⋯C contacts; see Fig. 5 ▸*b–f*. The HS analysis confirms the importance of H-atom contacts in establishing the packing (Hathwar *et al.*, 2015 ▸).

## 5. DFT Studies

The optimized structure of (**I**) in the gas phase was computed with *Gaussian09W* using the B3LYP/6–31G (d, p) basis set and generated by *GaussView5.0* (Frisch *et al.*, 2009 ▸). Comparison of experimentally determined bond lengths and angles (present single-crystal X-ray study) with those of theoretical values from the optimized structure showed good agreement [electronic supporting information (ESI), Table S1; the optimized mol­ecular structure of (**I**) is shown in ESI as Fig. S1].

**Figure 6:**
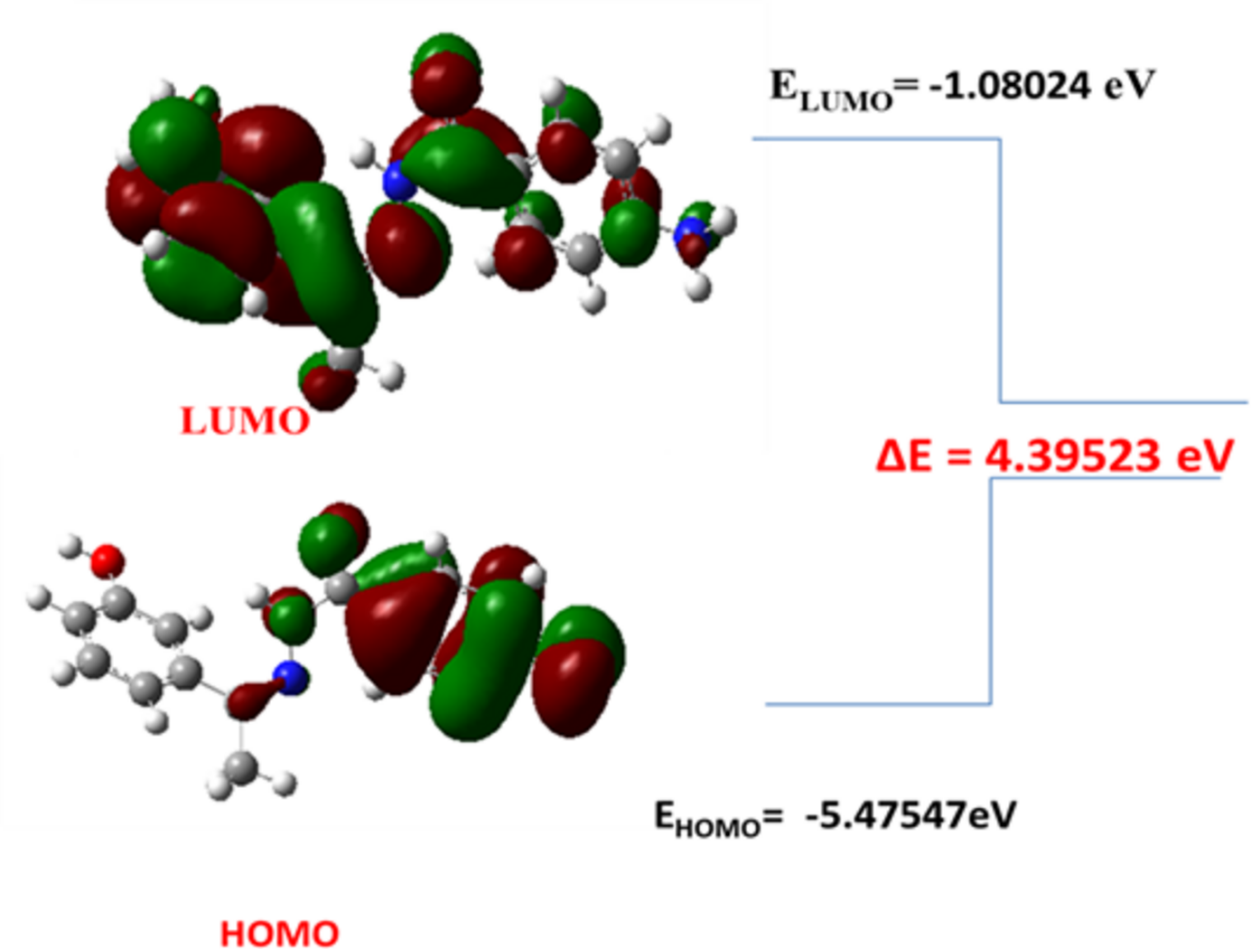
The HOMO/LUMO energy diagram of (**I**).

HOMO and LUMO (Fig. 6 ▸) were generated and their energies were evaluated from the optimized structure. The biological activity may also be comprehended by using the value of Δ*E* (Gulsevensidir *et al.*, 2011 ▸), which can be used to correlate and understand a decreased toxicity, longer half-life, and sustained activity. Therefore, it is anti­cipated that mol­ecule (**I**) with Δ*E* = 4.395 eV might have a strong biological influence with low adverse effects.

**Figure 7:**
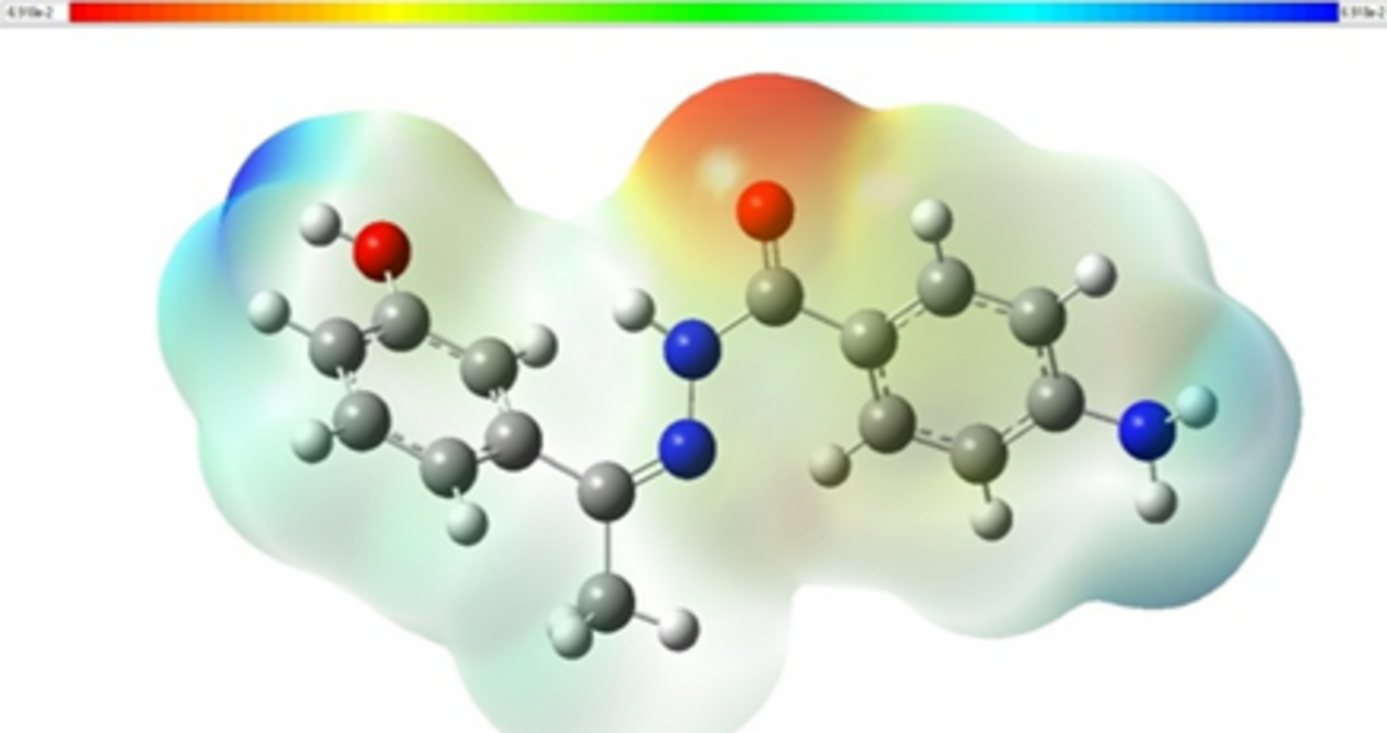
The mol­ecular electrostatic potential surface (MEPS) of (**I**).

The mol­ecular electrostatic potential surface (MEPS; Fig. 7 ▸) is used to find the positive and negative electrostatic potential of the mol­ecule, which provides possible information about its reactive sites with regard to chemical processes and binding sites for certain biological entities. The red-colored areas on the MEPS of (**I**) above the carbonyl oxygen atom of the azo­nitrile nitro­gen moiety, which is likely to undergo electrophilic attack, indicate the electron-rich portion with a partial negative charge. The mild-blue coloration of (**I**) suggests that there are slight electron-deficient regions. The lack of a bright-blue area on the MEPS indicates that the mol­ecule has no potential nucleophilic attack sites. The pale-blue color of the phenyl rings indicate weak electrophilic sites.

## 6. Synthesis and crystallization

4-Amino­benzohydrazide (2 mmol) and the corresponding substituted aromatic ketone (2 mmol) were dissolved in 25 ml of methanol, along with a few drops of acetic acid, to give a clear solution. The reaction mixture was filled in a round bottom flask and refluxed on a water bath for about 4 h. The progress of the reaction was monitored by thin layer chromatography (TLC). After completion of the reaction, methanol was removed by vacuum distillation. The solid product was collected, washed, and recrystallized from methanol to obtain a pure product of (**I**).

## 7. Refinement

Crystal data, data collection and structure refinement details are summarized in Table 2 ▸. Atom H2*A* was located from a difference-Fourier map; all other H atoms were placed in idealized positions and allowed to ride on their parent atoms with O—H = 0.82, N—H = 0.86 and C—H = 0.93–0.96 Å, respectively, and with *U*_iso_(H) = 1.5*U*_eq_(C) for methyl H atoms and *U*_iso_(H) = 1.2*U*_eq_(C)(C or N or O).

## Supplementary Material

Crystal structure: contains datablock(s) I, global. DOI: 10.1107/S205698902500297X/wm5754sup1.cif

Structure factors: contains datablock(s) I. DOI: 10.1107/S205698902500297X/wm5754Isup2.hkl

Table S1. DOI: 10.1107/S205698902500297X/wm5754sup3.docx

Figure S1. DOI: 10.1107/S205698902500297X/wm5754sup4.tif

Supporting information file. DOI: 10.1107/S205698902500297X/wm5754Isup5.cml

CCDC reference: 2203541

Additional supporting information:  crystallographic information; 3D view; checkCIF report

## Figures and Tables

**Table 1:** Hydrogen-bond geometry (Å, °)

*D*—H⋯*A*	*D*—H	H⋯*A*	*D*⋯*A*	*D*—H⋯*A*
O2—H2⋯O1^i^	0.82	1.88	2.694 (2)	171
N1—H1*B*⋯O1^ii^	0.86	2.13	2.958 (2)	162
N1—H1*A*⋯O2^iii^	0.86	2.37	3.119 (2)	146

Symmetry codes: (i) 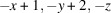; (ii) 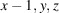; (iii) 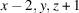.

**Table 2:** Experimental details

Crystal data	
Chemical formula	C_15_H_15_N_3_O_2_
*M*_r_	269.30
Crystal system, space group	Triclinic, *P* 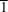
Temperature (K)	298
*a*, *b*, *c* (Å)	8.3562 (4), 9.2666 (4), 9.9151 (4)
α, β, γ (°)	76.685 (2), 65.316 (1), 84.909 (2)
*V* (Å^3^)	678.83 (5)
*Z*	2
Radiation type	Mo *K*α
μ (mm^−1^)	0.09
Crystal size (mm)	0.33 × 0.29 × 0.17

Data collection	
Diffractometer	Bruker D8 Quest XRD
No. of measured, independent and observed [*I* > 2σ(*I*)] reflections	13112, 3420, 2620
*R*_int_	0.026
(sin θ/λ)_max_ (Å^−1^)	0.707

Refinement	
*R*[*F*^2^ > 2σ(*F*^2^)], *wR*(*F*^2^), *S*	0.050, 0.134, 1.05
No. of reflections	3420
No. of parameters	186
No. of restraints	1
H-atom treatment	H atoms treated by a mixture of independent and constrained refinement
Δρ_max_, Δρ_min_ (e Å^−3^)	0.25, −0.15

Computer programs: *APEX3* and *SAINT* (Bruker, 2017 ▸), *SHELXT* (Sheldrick, 2015*a* ▸), *ORTEP-3 for Windows* (Farrugia, 2012 ▸) and *PLATON* (Spek, 2020 ▸), *SHELXL* (Sheldrick, 2015*b* ▸).

## supplementary crystallographic information

### 4-Amino-N'-[(1E)-1-(3-hydroxyphenyl)ethylidene]benzohydrazide Crystal data

**Table:**

C_15_H_15_N_3_O_2_	*Z* = 2
*M**_r_* = 269.30	*F*(000) = 284
Triclinic, *P*1	*D*_x_ = 1.318 Mg m^−^^3^
*a* = 8.3562 (4) Å	Mo *K*α radiation, λ = 0.71073 Å
*b* = 9.2666 (4) Å	Cell parameters from 5917 reflections
*c* = 9.9151 (4) Å	θ = 2.7–29.3°
α = 76.685 (2)°	µ = 0.09 mm^−^^1^
β = 65.316 (1)°	*T* = 298 K
γ = 84.909 (2)°	Block, colorless
*V* = 678.83 (5) Å^3^	0.33 × 0.29 × 0.17 mm

### 4-Amino-N'-[(1E)-1-(3-hydroxyphenyl)ethylidene]benzohydrazide Data collection

**Table:**

Bruker D8 Quest XRD diffractometer	*R*_int_ = 0.026
Detector resolution: 7.3910 pixels mm^-1^	θ_max_ = 30.2°, θ_min_ = 2.7°
ω and Phi Scans scans	*h* = −11→11
13112 measured reflections	*k* = −12→13
3420 independent reflections	*l* = −13→13
2620 reflections with *I* > 2σ(*I*)	

### 4-Amino-N'-[(1E)-1-(3-hydroxyphenyl)ethylidene]benzohydrazide Refinement

**Table:**

Refinement on *F*^2^	1 restraint
Least-squares matrix: full	Hydrogen site location: mixed
*R*[*F*^2^ > 2σ(*F*^2^)] = 0.050	H atoms treated by a mixture of independent and constrained refinement
*wR*(*F*^2^) = 0.134	*w* = 1/[σ^2^(*F*_o_^2^) + (0.0511*P*)^2^ + 0.2093*P*] where *P* = (*F*_o_^2^ + 2*F*_c_^2^)/3
*S* = 1.05	(Δ/σ)_max_ < 0.001
3420 reflections	Δρ_max_ = 0.25 e Å^−^^3^
186 parameters	Δρ_min_ = −0.15 e Å^−^^3^

### 4-Amino-N'-[(1E)-1-(3-hydroxyphenyl)ethylidene]benzohydrazide Special details

**Table:**

Geometry. All esds (except the esd in the dihedral angle between two l.s. planes) are estimated using the full covariance matrix. The cell esds are taken into account individually in the estimation of esds in distances, angles and torsion angles; correlations between esds in cell parameters are only used when they are defined by crystal symmetry. An approximate (isotropic) treatment of cell esds is used for estimating esds involving l.s. planes.

### 4-Amino-N'-[(1E)-1-(3-hydroxyphenyl)ethylidene]benzohydrazide Fractional atomic coordinates and isotropic or equivalent isotropic displacement parameters (Å^2^)

**Table:**

	*x*	*y*	*z*	*U*_iso_*/*U*_eq_	
O1	0.15425 (14)	0.90009 (13)	0.19913 (13)	0.0525 (3)	
O2	0.99259 (14)	0.84728 (15)	−0.28575 (16)	0.0642 (4)	
H2	0.939270	0.922396	−0.261740	0.096*	
N1	−0.67521 (18)	0.83831 (19)	0.41445 (18)	0.0646 (4)	
H1A	−0.730474	0.838506	0.509568	0.078*	
H1B	−0.733195	0.836403	0.360773	0.078*	
N2	0.14917 (16)	0.77285 (15)	0.03196 (16)	0.0470 (3)	
N3	0.33033 (15)	0.76091 (14)	−0.03496 (14)	0.0449 (3)	
C1	−0.22296 (19)	0.83740 (17)	0.12941 (16)	0.0432 (3)	
H1	−0.164218	0.833788	0.027285	0.052*	
C2	−0.4043 (2)	0.83844 (17)	0.19445 (17)	0.0456 (3)	
H2B	−0.466420	0.837429	0.135719	0.055*	
C3	−0.49542 (19)	0.84101 (17)	0.34866 (18)	0.0449 (3)	
C4	−0.3979 (2)	0.84413 (19)	0.43329 (17)	0.0490 (4)	
H4	−0.456202	0.844926	0.536114	0.059*	
C5	−0.2173 (2)	0.84603 (18)	0.36659 (17)	0.0465 (4)	
H5	−0.155097	0.850317	0.424490	0.056*	
C6	−0.12531 (18)	0.84166 (16)	0.21355 (16)	0.0395 (3)	
C7	0.06932 (19)	0.84259 (16)	0.14849 (17)	0.0413 (3)	
C8	0.38915 (19)	0.66826 (16)	−0.12234 (17)	0.0430 (3)	
C9	0.2747 (2)	0.5749 (3)	−0.1523 (3)	0.0787 (7)	
H9A	0.347537	0.514173	−0.221098	0.118*	
H9B	0.199464	0.512823	−0.058500	0.118*	
H9C	0.204057	0.638171	−0.196627	0.118*	
C10	0.58360 (19)	0.65143 (16)	−0.19348 (16)	0.0406 (3)	
C11	0.69503 (19)	0.76129 (16)	−0.20501 (16)	0.0417 (3)	
H11	0.647841	0.846282	−0.168357	0.050*	
C12	0.87624 (19)	0.74403 (17)	−0.27108 (17)	0.0449 (3)	
C13	0.9479 (2)	0.61656 (19)	−0.32493 (18)	0.0488 (4)	
H13	1.069377	0.604830	−0.368656	0.059*	
C14	0.8379 (2)	0.50825 (18)	−0.31303 (19)	0.0507 (4)	
H14	0.885554	0.422900	−0.348736	0.061*	
C15	0.6570 (2)	0.52448 (17)	−0.24861 (19)	0.0495 (4)	
H15	0.584065	0.450565	−0.242003	0.059*	
H2A	0.082 (2)	0.730 (2)	0.005 (2)	0.068 (6)*	

### 4-Amino-N'-[(1E)-1-(3-hydroxyphenyl)ethylidene]benzohydrazide Atomic displacement parameters (Å^2^)

**Table:**

	*U*^11^	*U*^22^	*U*^33^	*U*^12^	*U*^13^	*U*^23^
O1	0.0408 (6)	0.0658 (7)	0.0606 (7)	0.0009 (5)	−0.0226 (5)	−0.0283 (6)
O2	0.0385 (6)	0.0698 (8)	0.0813 (9)	−0.0062 (5)	−0.0078 (6)	−0.0407 (7)
N1	0.0364 (7)	0.0989 (12)	0.0575 (9)	−0.0049 (7)	−0.0141 (6)	−0.0225 (8)
N2	0.0338 (6)	0.0549 (7)	0.0551 (8)	−0.0020 (5)	−0.0144 (6)	−0.0237 (6)
N3	0.0333 (6)	0.0503 (7)	0.0497 (7)	−0.0023 (5)	−0.0125 (5)	−0.0159 (6)
C1	0.0427 (8)	0.0501 (8)	0.0379 (7)	−0.0027 (6)	−0.0150 (6)	−0.0128 (6)
C2	0.0427 (8)	0.0545 (9)	0.0457 (8)	−0.0019 (6)	−0.0219 (6)	−0.0139 (7)
C3	0.0369 (7)	0.0491 (8)	0.0473 (8)	−0.0025 (6)	−0.0150 (6)	−0.0108 (7)
C4	0.0442 (8)	0.0635 (10)	0.0363 (7)	0.0002 (7)	−0.0132 (6)	−0.0110 (7)
C5	0.0429 (8)	0.0586 (9)	0.0420 (8)	0.0002 (7)	−0.0201 (6)	−0.0128 (7)
C6	0.0361 (7)	0.0415 (7)	0.0411 (7)	−0.0018 (5)	−0.0146 (6)	−0.0108 (6)
C7	0.0381 (7)	0.0416 (7)	0.0448 (8)	−0.0014 (6)	−0.0169 (6)	−0.0100 (6)
C8	0.0406 (7)	0.0448 (7)	0.0455 (8)	−0.0011 (6)	−0.0173 (6)	−0.0133 (6)
C9	0.0488 (10)	0.0951 (15)	0.1132 (18)	0.0044 (10)	−0.0324 (11)	−0.0646 (14)
C10	0.0405 (7)	0.0435 (7)	0.0380 (7)	−0.0002 (6)	−0.0148 (6)	−0.0114 (6)
C11	0.0403 (7)	0.0446 (7)	0.0395 (7)	0.0009 (6)	−0.0121 (6)	−0.0163 (6)
C12	0.0403 (8)	0.0535 (8)	0.0409 (7)	−0.0024 (6)	−0.0120 (6)	−0.0181 (7)
C13	0.0397 (8)	0.0589 (9)	0.0464 (8)	0.0053 (7)	−0.0122 (6)	−0.0210 (7)
C14	0.0525 (9)	0.0457 (8)	0.0519 (9)	0.0074 (7)	−0.0159 (7)	−0.0198 (7)
C15	0.0503 (9)	0.0441 (8)	0.0542 (9)	−0.0023 (6)	−0.0176 (7)	−0.0168 (7)

### 4-Amino-N'-[(1E)-1-(3-hydroxyphenyl)ethylidene]benzohydrazide Geometric parameters (Å, º)

**Table:**

O1—C7	1.2360 (17)	C5—C6	1.394 (2)
O2—C12	1.3656 (18)	C5—H5	0.9300
O2—H2	0.8200	C6—C7	1.4782 (19)
N1—C3	1.3652 (19)	C8—C10	1.487 (2)
N1—H1A	0.8600	C8—C9	1.501 (2)
N1—H1B	0.8600	C9—H9A	0.9600
N2—C7	1.3496 (19)	C9—H9B	0.9600
N2—N3	1.3818 (17)	C9—H9C	0.9600
N2—H2A	0.864 (9)	C10—C11	1.394 (2)
N3—C8	1.2811 (19)	C10—C15	1.395 (2)
C1—C2	1.377 (2)	C11—C12	1.387 (2)
C1—C6	1.397 (2)	C11—H11	0.9300
C1—H1	0.9300	C12—C13	1.392 (2)
C2—C3	1.400 (2)	C13—C14	1.373 (2)
C2—H2B	0.9300	C13—H13	0.9300
C3—C4	1.399 (2)	C14—C15	1.383 (2)
C4—C5	1.371 (2)	C14—H14	0.9300
C4—H4	0.9300	C15—H15	0.9300
			
C12—O2—H2	109.5	N2—C7—C6	115.58 (12)
C3—N1—H1A	120.0	N3—C8—C10	116.54 (13)
C3—N1—H1B	120.0	N3—C8—C9	124.22 (14)
H1A—N1—H1B	120.0	C10—C8—C9	119.22 (13)
C7—N2—N3	121.26 (12)	C8—C9—H9A	109.5
C7—N2—H2A	117.2 (13)	C8—C9—H9B	109.5
N3—N2—H2A	121.4 (13)	H9A—C9—H9B	109.5
C8—N3—N2	115.54 (12)	C8—C9—H9C	109.5
C2—C1—C6	121.41 (13)	H9A—C9—H9C	109.5
C2—C1—H1	119.3	H9B—C9—H9C	109.5
C6—C1—H1	119.3	C11—C10—C15	119.07 (14)
C1—C2—C3	120.25 (14)	C11—C10—C8	120.63 (13)
C1—C2—H2B	119.9	C15—C10—C8	120.30 (13)
C3—C2—H2B	119.9	C12—C11—C10	120.09 (13)
N1—C3—C4	121.19 (14)	C12—C11—H11	120.0
N1—C3—C2	120.41 (14)	C10—C11—H11	120.0
C4—C3—C2	118.39 (14)	O2—C12—C11	123.02 (13)
C5—C4—C3	120.85 (14)	O2—C12—C13	116.66 (13)
C5—C4—H4	119.6	C11—C12—C13	120.32 (14)
C3—C4—H4	119.6	C14—C13—C12	119.50 (14)
C4—C5—C6	121.19 (14)	C14—C13—H13	120.3
C4—C5—H5	119.4	C12—C13—H13	120.2
C6—C5—H5	119.4	C13—C14—C15	120.83 (14)
C5—C6—C1	117.89 (13)	C13—C14—H14	119.6
C5—C6—C7	118.67 (13)	C15—C14—H14	119.6
C1—C6—C7	123.44 (13)	C14—C15—C10	120.19 (14)
O1—C7—N2	121.81 (13)	C14—C15—H15	119.9
O1—C7—C6	122.59 (13)	C10—C15—H15	119.9
			
C7—N2—N3—C8	166.52 (15)	N2—N3—C8—C10	−178.92 (13)
C6—C1—C2—C3	−1.2 (2)	N2—N3—C8—C9	−0.7 (2)
C1—C2—C3—N1	−178.36 (15)	N3—C8—C10—C11	−20.1 (2)
C1—C2—C3—C4	0.7 (2)	C9—C8—C10—C11	161.65 (17)
N1—C3—C4—C5	179.65 (16)	N3—C8—C10—C15	159.42 (15)
C2—C3—C4—C5	0.6 (2)	C9—C8—C10—C15	−18.9 (2)
C3—C4—C5—C6	−1.4 (3)	C15—C10—C11—C12	0.3 (2)
C4—C5—C6—C1	0.9 (2)	C8—C10—C11—C12	179.74 (13)
C4—C5—C6—C7	−179.44 (14)	C10—C11—C12—O2	−179.94 (15)
C2—C1—C6—C5	0.4 (2)	C10—C11—C12—C13	−0.6 (2)
C2—C1—C6—C7	−179.23 (14)	O2—C12—C13—C14	179.78 (15)
N3—N2—C7—O1	0.9 (2)	C11—C12—C13—C14	0.4 (2)
N3—N2—C7—C6	−177.49 (13)	C12—C13—C14—C15	0.2 (3)
C5—C6—C7—O1	−27.6 (2)	C13—C14—C15—C10	−0.5 (3)
C1—C6—C7—O1	152.05 (15)	C11—C10—C15—C14	0.3 (2)
C5—C6—C7—N2	150.74 (15)	C8—C10—C15—C14	−179.20 (14)
C1—C6—C7—N2	−29.6 (2)		

### 4-Amino-N'-[(1E)-1-(3-hydroxyphenyl)ethylidene]benzohydrazide Hydrogen-bond geometry (Å, º)

**Table:**

*D*—H···*A*	*D*—H	H···*A*	*D*···*A*	*D*—H···*A*
O2—H2···O1^i^	0.82	1.88	2.694 (2)	171
N1—H1*B*···O1^ii^	0.86	2.13	2.958 (2)	162
N1—H1*A*···O2^iii^	0.86	2.37	3.119 (2)	146

Symmetry codes: (i) −*x*+1, −*y*+2, −*z*; (ii) *x*−1, *y*, *z*; (iii) *x*−2, *y*, *z*+1.
