# Supplementary material for: Crystal structure, Hirshfeld surface analysis and DFT studies of 4-amino-N′-[(1E)-1-(3-hy­droxyphen­yl)ethyl­idene]benzohydrazide
Source: Acta Crystallogr E Crystallogr Commun. 2025 Apr 8;81(Pt 5):389–92. doi: 10.1107/S205698902500297X (PMC12054771; doi:10.1107/S205698902500297X)
Supplement: Supplementary file 3 [file e-81-00389-sup3.docx]

**Comparison (X-ray and DFT) of selected bond lengths and bond angles (Å,°).**

| **Parameter** | **Single Crystal-XRD** | **B3LYP/6–31G(d,p)** |
| --- | --- | --- |
| O1-C7 | 1.2360(17) | 1.2584 |
| O2-C12 | 1.3656(18) | 1.43 |
| N1-C3 | 1.3652(19) | 1.47 |
| N2-C7 | 1.3496(19) | 1.47 |
| N2-N3 | 1.3818(17) | 1.3998 |
| N3-C8 | 1.2811(19) | 1.2938 |
| C1-C2 | 1.377(2) | 1.3947 |
| C1-C6 | 1.397(2) | 1.3952 |
| C2-C3 | 1.400(2) | 1.3954 |
| C3-C4 | 1.399(2) | 1.3998 |
| C4-C5 | 1.371(2) | 1.3961 |
| C5-C6 | 1.394(2) | 1.3948 |
| C6-C7 | 1.4782(19) | 1.54 |
| C8-C10 | 1.487(2) | 1.54 |
| C8-C9 | 1.501(2) | 1.54 |
| C10-C11 | 1.394(2) | 1.3952 |
| C10-C15 | 1.395(2) | 1.3949 |
| C11-C12 | 1.387(2) | 1.3948 |
| C12-C13 | 1.392(2) | 1.3954 |
| C13-C14 | 1.373(2) | 1.3947 |
| C14-C15 | 1.383(2) | 1.3952 |
| C7-N2-N3 | 121.26(12) | 109.4769 |
| C2-C1-C6 | 121.41(13) | 120.0083 |
| C1-C2-C3 | 120.25(14) | 119.994 |
| N1-C3-C4 | 121.19(14) | 120.025 |
| N1-C3-C2 | 120.41(14) | 119.981 |
| C4-C3-C2 | 118.39(14) | 119.994 |
| C5-C4-C3 | 120.85(14) | 120.0049 |
| C4-C5-C6 | 121.19(14) | 119.9997 |
| C5-C6-C1 | 117.89(13) | 119.9991 |
| C5-C6-C7 | 118.67(13) | 120.0031 |
| C1-C6-C7 | 123.44(13) | 119.9978 |
| O1-C7-N2 | 121.81(13) | 120.2262 |
| O1-C7-C6 | 122.59(13) | 119.8869 |
| N2-C7-C6 | 115.58(12) | 120.2262. |
| N3-C8-C10 | 116.54(13) | 120.2227 |
| N3-C8-C9 | 124.22(14) | 119.8875 |
| C10-C8-C9 | 119.22(13) | 119.9889 |
| C11-C10-C15 | 119.07(14) | 119.9889 |
| C11-C10-C8 | 120.63(13) | 119.9889 |
| C15-C10-C8 | 120.30(13) | 120.01 |
| C12-C11-C10 | 120.09(13) | 119.9937 |
| O2-C12-C11 | 123.02(13) | 120.0232 |
| O2-C12-C13 | 116.66(13) | 119.9832 |
| C11-C12-C13 | 120.32(14) | 119.9937 |
| C14-C13-C12 | 119.50(14) | 119.997 |
| C13-C14-C15 | 120.83(14) | 120.008 |
| C14-C15-C10 | 120.19(14) | 119.9955 |
