# Supplementary figures and images for: Crystal structure, Hirshfeld surface analysis and DFT studies of 4-amino-N′-[(1E)-1-(3-hy­droxyphen­yl)ethyl­idene]benzohydrazide
Source: Acta Crystallogr E Crystallogr Commun. 2025 Apr 8;81(Pt 5):389–92. doi: 10.1107/S205698902500297X (PMC12054771; doi:10.1107/S205698902500297X)

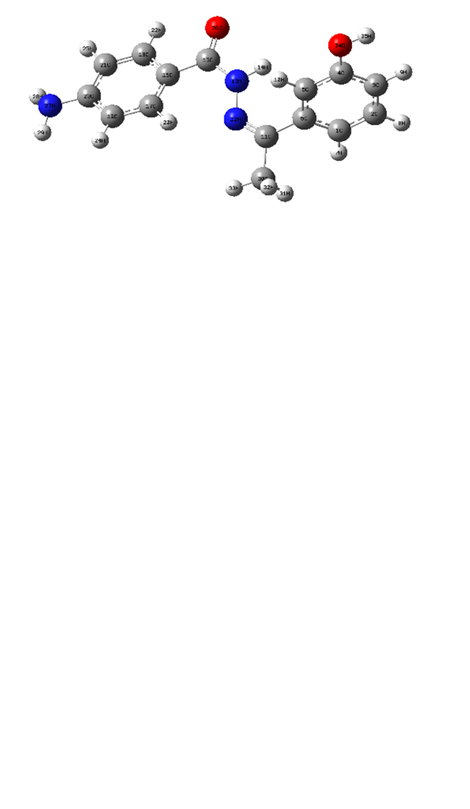

Supplement: Supplementary file 4 [file e-81-00389-sup4.tif]
